# Metallic Nanoparticles and Core-Shell Nanosystems in the Treatment, Diagnosis, and Prevention of Parasitic Diseases

**DOI:** 10.3390/pathogens12060838

**Published:** 2023-06-17

**Authors:** Grzegorz Król, Kamila Fortunka, Michał Majchrzak, Ewelina Piktel, Paulina Paprocka, Angelika Mańkowska, Agata Lesiak, Maciej Karasiński, Agnieszka Strzelecka, Bonita Durnaś, Robert Bucki

**Affiliations:** 1 Department of Microbiology and Immunology, Institute of Medical Science, Collegium Medicum, Jan Kochanowski University, IX Wieków Kielc 19A, 25-317 Kielce, Poland; kamilafortunka@gmail.com (K.F.); fulgryym@gmail.com (M.M.); paulina.paprocka@ujk.edu.pl (P.P.); angelika.mankowska@ujk.edu.pl (A.M.); agatalesiak@wp.pl (A.L.); bonita.durnas@ujk.edu.pl (B.D.); buckirobert@gmail.com (R.B.); 2 Independent Laboratory of Nanomedicine, Medical University of Białystok, Mickiewicza 2B, 15-222 Białystok, Poland; ewelina.piktel@umb.edu.pl; 3 Department of Medical Microbiology and Nanobiomedical Engineering, Medical University of Białystok, Mickiewicza 2C, 15-222 Białystok, Poland; maciek.karasinski@gmail.com; 4 Department of Public Health , Institute of Health Science, Collegium Medicum, Jan Kochanowski University, IX Wieków Kielc 19A, 25-317 Kielce, Poland; agnieszka.strzelecka@ujk.edu.pl

**Keywords:** nanotechnology, metallic nanoparticles, nanosystems, parasitology

## Abstract

The usage of nanotechnology in the fight against parasitic diseases is in the early stages of development, but it brings hopes that this new field will provide a solution to target the early stages of parasitosis, compensate for the lack of vaccines for most parasitic diseases, and also provide new treatment options for diseases in which parasites show increased resistance to current drugs. The huge physicochemical diversity of nanomaterials developed so far, mainly for antibacterial and anti-cancer therapies, requires additional studies to determine their antiparasitic potential. When designing metallic nanoparticles (MeNPs) and specific nanosystems, such as complexes of MeNPs, with the shell of attached drugs, several physicochemical properties need to be considered. The most important are: size, shape, surface charge, type of surfactants that control their dispersion, and shell molecules that should assure specific molecular interaction with targeted molecules of parasites’ cells. Therefore, it can be expected that the development of antiparasitic drugs using strategies provided by nanotechnology and the use of nanomaterials for diagnostic purposes will soon provide new and effective methods of antiparasitic therapy and effective diagnostic tools that will improve the prevention and reduce the morbidity and mortality caused by these diseases.

## 1. Introduction

Despite continuous improvement of antiparasitic therapies, statistics show that parasites are responsible for about 14 million deaths per year and the total number of people affected by parasites is close to 3.5 billion [1]. Parasites have developed a wide range of potential transmission routes, from which it is difficult to determine which is of the greatest epidemiological importance. In the course of evolution, there have been many adaptations to specific forms of parasitism, including the ability to modulate the host’s immune response, high fecundity of parasites with long survival of invasive forms, as well as the use of parasitic vectors [2,3,4,5]. In humans, parasitic infections most often occur as a result of drinking contaminated water (e.g., *Cryptosporidium parvum*), consumption of raw or undercooked food (e.g., *Giardia intestinalis*, *Diphyllobothrium latum*, and *Trichinella spiralis*), inhalation of air with dust that can serve as a mean of transport for the parasites’ eggs (e.g., *Enterobius vermicularis*), contact with animals (e.g., *Toxocara* spp. or *Diphylidium caninum*), or contact with animal excrements (e.g., infection caused by *Echinococcus multilocularis*, and *Toxoplasma gondii*) [5,6,7,8,9,10,11,12,13,14]. An additional factor that contributes to parasite infections is travel to tropical areas, which often increases the chance of acquiring parasites, such as *Schistosoma haematobium*, *Schistosoma mansoni*, *Plasmodium* spp., and *Trypanosoma* spp. [15,16,17,18]. Sexual contact can also lead to infections caused by *Trichomonas vaginalis* or *Pthirus pubis* [19,20,21]. Nowadays parasitic diseases are a growing health problem in developing countries. Some of these diseases, such as leishmaniasis, African trypanosomiasis, or Chagas’ disease, belong to the group of so-called “neglected tropical diseases” (NTDs) which are prevalent in tropical and subtropical countries [22,23]. Therapeutic agents used to treat these diseases have limited efficacy and cause harmful or, sometimes, potentially fatal side-effects [24,25]. An additional problem of antiparasitic drugs (especially when administered against intracellular parasites) is their poor penetration through biological membranes of tissues and cells, which results in low bioavailability and, in consequence, lack of therapeutic effect [26,27,28]. These limitations might be greatly addressed by drugs in the form of nanosystems as they are usually characterized by increased tissue/cell penetration and high biocompatibility. The main ways of action of antiparasitic drugs are: blockade of parasite neuromuscular functions (causing muscle paralysis), induction of apoptosis, and inhibition of DNA and RNA synthesis. Antiprotozoal agents usually target the rapidly proliferating, cells. They also might target the synthesis of proteins or the specific metabolic pathways that distinguish host and parasite signal transmission pathways [27]. Standard treatment of parasitic diseases, apart from surgical removal of the parasite, is mainly based on the administration of one or a combination of few antiparasitic drugs (especially in the case of drug-resistance). The combination of several drugs is widely used. For example, in malaria a usual treatment is chloroquine combined with sulfadoxine–pyrimethamine [27] or, in the case of resistant strains, a combination of quinine and doxycycline or clindamycin. Such combinations often cause high toxicity, so new treatments are urgently needed. The drugs used in selected groups of parasites are listed in Table 1 [29,30,31].

One possible future solution to improve management of parasitic disease such as malaria may be the application of nanomaterials, such as metallic nanoparticles. Inorganic nanoparticles may be toxic. Factors affecting the cytotoxicity of metallic nanoparticles include, but are not limited to, size, shape, surface charge, nanoparticle synthesis, and metal ion release. This toxicity can be modulated by changing the shape and size of the particles and modifying their surface, thanks to which we can obtain nanoparticles with the desired properties, and controlled toxicity [27,32]. An increasing number of studies confirm this potential [27,33,34,35,36,37,38,39,40,41,42,43,44,45,46,47,48]. Currently, a significant number of nanosystems developed for medical use are composed of metallic nanoparticles with drug molecules attached to their surface, along with lipid particles or additional polymers. They can specifically affect tissues and cells by direct binding to specific receptors on the cell surface, which may be considered as their targets. They can also diffuse, accumulate within the tissue, and induce/provide some intrinsic and synergistic activity [24,49]. Their intrinsic properties, such as interaction with target cells, circulation time, renal clearance, protein absorption, and toxicity, can be governed by particle size, shape, diameter, and surface properties [35]. MeNPs can be synthetized using a wide spectrum of metals and metal oxides, such as gold, silver, iron oxide, copper oxide, zinc oxide, aluminium oxide, titanium dioxide, or gallium [50,51].

## 2. Different Strategies for Using MNPs and MNPs-Containing Nanosystems in Diagnostics, Prevention, and Therapy of Selected Parasitic Diseases

In the human body, parasites produce various chemical compounds and induce different physical effects, usually causing mechanical damage to tissues or organs. This process can be transient (reversible), but also permanent—such as liver or bladder damage occurring, respectively, during *Echinoccocus multilocularis* and *Schisthosoma haemathobium* infection. Some infections can directly cause the death of the host, such as neurocysticosis (*Taenia solium*), but, on the other hand, some parasites may not cause any symptoms, camouflaging themselves in the human body. Their presence might be revealed only in certain clinical conditions, such as immunosuppression or cancer (e.g., *Toxoplasma gondii*, and *Cryptosporidium parvum*) [12,52,53,54,55,56].

The use of nanotechnology can offer in the future many unique opportunities for better diagnostics of parasitic diseases. Diagnostic tools used nowadays for the detection of parasitic diseases include light and fluorescence microscopy, rapid diagnostic tests (RDT), such as immunochromatographic lateral flow tests, serology, quantitative Buffy-coat (QBC) techniques, and nucleic acid amplification techniques (mainly polymerase chain reaction—PCR) [44,57,58,59]. The diagnostics might be improved by the development of novel methods which use nanomaterials. Fluorescent, magnetic, and metal nanoparticles are currently being tested for their diagnostic potential. Among the latter, gold and silver nanoparticles are the most studied. They can emit intense absorption when excited by electromagnetic radiation [60]. Various molecules, such as antibodies, antigens, and enzymes, can be conjugated to nanoparticles as electrochemical markers, optical probes, and signal enhancers. An example is the use of magnetic nanoparticles with an iron oxide core and a silver coating for the early detection of malaria [61,62].

In the treatment of parasitosis, the key challenge is precise drug delivery to assure parasite elimination with low toxicity to host tissues and cells. The presence of specific homing ligands attached to the surface of the nanosystem might potentially allow selective binding to the parasite cell receptors, guaranteeing precise drug delivery [63,64,65]. Usage of MeNPs for drug delivery requires drugs attachment to nanoparticles surface that can be achieved by chemical adsorption, encapsulation, and conjugation. MeNPs and nanosystem administration can be conducted by oral, intragastric, intravenous, intraduodenal, pulmonary, and cutaneous methods [27,66,67,68,69]. Metal-based nanoparticles for biomedical applications need to meet several requirements such as stability, lack of aggregation, host biocompatibility, and cell/tissue selectivity [35,43,70,71]. There are experimental methods of usage of MeNPs to diagnose and treat cancers which display the theranostic potential of nanoparticles [72]. Parasitic diseases can interfere with, promote, or modulate carcinogenesis. Some parasites can modify the host’s immune response, thereby modifying the tumor microenvironment. For example, studies conducted in the case of active infection with *Toxoplasma gondii* in mice challenged with Lewis lung carcinoma cells showed increased survival. Similar studies resulted from the use of *Plasmodium* sp. Inducing strong innate and adaptive anti-cancer immune responses promoted survival and reduced the proliferation of Lewis lung cancer cells in an experimental model [73]. A separate group are parasites affecting the development of cancer, which include, among others: *Schistosoma haematobium*—associated with the development of bladder cancer; *Schistosoma mansoni* in people infected with hepatitis C virus (HCV)—increased risk of hepatocellular carcinoma; and *Clonorchis synesis*, the parasite that causes clonorchiasis, has been classified as highly carcinogenic, causing bile-duct carcinoma [73].

Magnetic hyperthermia using MNP comprises locally increasing the temperature above 42 °C, influencing the physiology of cancer cells by changing structural proteins, and inducing cell necrosis or apoptosis. In some parasitic diseases, there are intermediate forms in the form of cysts, e.g., *Echinococcus granulosus*, and *Taenia multiceps*. It can be assumed that the use of nanoparticles to induce hyperthermia, as in the case of cancer, can potentially be used in the fight against parasitic diseases in which one of the developmental forms is cysts. It might be supposed that this phenomenon, as in case of cancers, can potentially be used to fight parasitic diseases, especially those associated with organ tumor formation [50,51,74].

**Figure 1:**
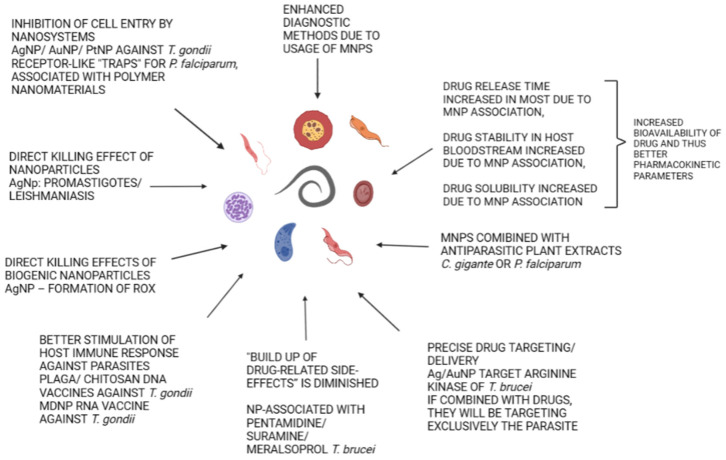
Possible usage of nanosystems in antiparasitic treatment.

Recently, the development of new therapeutic strategies with the use of nanomaterials has been proposed for different parasitic diseases. A large number of studies were dedicated to leishmaniasis, African trypanosomiasis, American trypanosomiasis, malaria, toxoplasmosis, and schistosomiasis (see Figure 1, Table 2) [28,75,76].

### 2.1. Leishmaniasis

Leishmaniasis is a disease caused by a parasitic protozoan from the family of *Trypanosomatidae*. More than 20 species of leishmaniasis infect humans and the vector for it are female flies. The disease affects over 10 million people in about 90 tropical and subtropical countries. The WHO reports that the number of people at risk of leishmaniasis is ~350 million [98]. The most life-threatening form is visceral leishmaniasis (about 400,000 cases per year—95% mortality in untreated cases) [24,99]. The most common form is cutaneous leishmaniasis, which affects approximately 2 million people annually. It causes many clinical symptoms, including lumps and skin ulcers [99,100,101].

Many research groups have focused on development of novel approaches to treat and diagnose leishmaniasis. These efforts in the future can result in development of several potential drugs that are based on metallic nanoparticle. A considerable effectiveness was reported for silver nanoparticles (AgNPs) that cause damage to the structural integrity of *Leishmania amazniensis* amastigotes. AgNPs lead to the death of promastigotes and damage to amastigotes. The effect is a reduced number of infected macrophages, reduction of their metabolic activity, reduction of proliferation, and hastened deterioration [64,81,82,102,103]. Zinc oxide nanoparticles (ZnONPs) seem to be also effective. In in vitro studies, they display antiparasitic properties against *Leishmania tropica* [104]. Similarly, ZnONPs exert cytotoxicity against amastigote cells affecting the proliferation and metabolic activity of *Leishmania donovanii*. Interestingly, extracts of *Verbena officinalis* and *Verbena tenuisecta* might potentialize ZnONPs activity against the *Leishmania* parasite [85,102].

Gold nanoparticles conjugated with quercetin (QAuNPs) have also shown effectiveness and selectivity against *Leishmania’s* amastigote. Promising results were obtained when nanoparticles were used together with ultraviolet (UV) and infrared (IR) radiation—this enhances the killing effect of the nanoparticles, associated with increased generation of reactive oxygen species. While testing anti-leishmatic activity, UV and IR radiation effects were also assessed in the presence of silver nanoparticles (AgNPs), gold nanoparticles (AuNPs), titanium dioxide nanoparticles (TiO_2_NPs), zinc oxide nanoparticles (ZnONP), and magnesium oxide nanoparticles (MgONPs). All these experiments have shown promising anti-parasite effects, which was the most effective in case of silver and gold-based nanomaterials. However, when combined with UV and IR radiation, most of metallic nanoparticles displayed high toxicity to macrophages [80]. 

One possible way to address the high toxicity to macrophages might be the use of biogenic silver and gold nanoparticles. It was found that biogenic AgNPs are more efficient than those chemically synthesized—they have a similar effect on *Leishmania* at lower concentrations. They induce reactive oxygen species formation more strongly, resulting in the death of amastigotes [105,106]. It is worth underlining that biogenously synthesized AgNPs inhibit the growth of the parasite in a concentration-dependent manner. The effectiveness of biogenic silver nanoparticles (extract from *Moringa oleifera*, a horseradish tree) against leishmaniasis is worth pointing out due to their high efficiency. Indeed, the synthesis of AgNP using fungi, bacteria, or plants should be considered as an innovative approach in an attempt to reduce MNPs’ toxicity. Additionally, bioparticles conjugated with enzymes, proteins, polysaccharides, or amino acids that are present in plant extracts might have different, and usually better, therapeutic value when compared to those synthesized by stabilizing and reducing silver ions. Apart from microorganisms, biosynthesis of metallic nanomaterials can be achieved using plant species, such as *Coriandrum sativum*, *Azadirachta indica*, *Nelumbo nucifera*, *Ocimum basilicum*, and several others [107,108,109].

### 2.2. African Trypanosomiasis (African Coma)

Trypanosomes are a type of parasitic protozoan characterized by the presence of a single strand of dense DNA called a kinetoplast. They are responsible, inter alia, for trypanosomiasis (*Trypanosoma brucei*) and the American–Chagas’ disease (*Trypanosoma cruzi*). Infectious forms of *Trypanosoma cruzi* are trypomastigotes that can infect many different cell types through a process independent of the host cell’s actin polymerization. Parasite adhesion is driven by particles present on the surface of the parasite or secreted during the infection process; these molecules bind to specific receptors on the host cells [110]. The human African trypanosomiasis is caused by the protozoan *Trypanosoma brucei gambiense* (milder form) and *Trypanosoma brucei rhodesiense* (associated with faster disease progression) that involve development of neuronal symptoms and higher mortality [30,111]. The disease occurs only in Sub-Saharan Africa and is transmitted by vectors—the flies of genus *Glossina* [112,113,114]. When untreated, the disease often leads to coma and death due to multi-organ failure. Estimated data show that 65 million people living in the 36 Sub-Saharan African countries are at risk of contracting the disease [113]. Production of an effective vaccine is difficult due to the presence of a parasitic coat with variable surface glycoprotein (VSG) [24]. It allows trypanosomatic parasites to evade the host’s immune system through extensive antigenic variability [24,113]. Currently, drugs used to treat the infection cause serious adverse effects that were summarized in Table 1. The use of metal nanoparticles as a drug-delivery system for the above-mentioned compounds can, potentially, significantly reduce the occurrence of side-effects. Combinations of nanoparticles and drugs might have higher efficacy compared to anti-pathogenic agents alone, and can shorten the duration and doses of the drug administered. Functionalization of metallic nanoparticles with anti-pathogenic agents might also improve the pharmacokinetic properties of the drug by increasing its solubility, extending the half-life of the drug and the time of systemic circulation, which might result in lower doses and frequency of administration of the drug, thus reducing its cytotoxicity [27,115,116,117]. Metallic nanoparticles, such as silver and gold, display different potential against different species of *Trypanosoma* (*Trypanosoma brucei*, *Trypanosoma congolense*, and *Trypanosoma evansi*). Gold nanoparticles were less effective against *Trypanosoma congolense* and *Trypanosoma evansi*, with an average ≤50% inhibition of parasite growth compared to silver nanoparticles, which were ≥70%. Gold and silver nanoparticles strongly and selectively inhibit recombinant *Trypanosoma brucei* arginine kinase [77,111]. Arginine kinase is a phosphotransferase that is essential for the growth and survival of *Trypanosoma brucei,* particularly in the bloodstream of the infected host because it helps the parasite meet required energy demands to maintain a reductive environment [24,77]. The high selectivity of the nanoparticles for *Trypanosoma* spp.-specific kinase indicates a high potential for combining them with drugs.

### 2.3. Chagas’ Disease, American Trypanosomiasis

Chagas’ disease is caused by *Trypanosoma cruzi.* It is associated with a high level of morbidity and mortality, and its treatment is often not effective due to drug toxicity and low bioavailability [118]. The disease affects about 8 million people and causes about 50,000 deaths per year [22,119]. The disease can be transmitted perinatally; 15,000 newborns are born as infected each year [120,121]. The number of infected women of childbearing age is about 1 million and the risk of transmitting the parasite to the child is 3 to 5% [22]. Some recent Chagas’ disease diagnostics methods involved the use of nanomaterials—including metallic nanoparticles. More precisely, these diagnostic tools should be used in an integrated microflow system connected to a carbon-screen-printed electrode (SPCE) [122]. It is designed to quantify specific IgG antibodies in serum. It uses AuNPs functionalized with *Trypanosoma cruzi* proteins isolated from epimastigotes. The lower detection limit of the test was established to be 3.065 ng/mL and the coefficients of variation within and between tests were lower than 6.95% [122]. For early diagnosis of congenital Chagas’ disease, the Chunap test (Chagas’ urine nanoparticles test) is used. It is based on the Western blot detection of antigens that were secreted by trypomastigote forms [123]. The sensitivity and specificity of a urine-based test are, approximately, 90 and 95%, respectively, due to the use of nanoparticles that have been functionalized with polyunsaturated N-isopropylacrylamide and tryptan blue that can capture and concentrate *T. cruzi* [122]. Nifurtimox and benzimidazole are used in the treatment of Chagas’ disease. They are mainly associated with dermatological side-effects, fever, and lymphadenopathy [22,124]. Rarely, such conditions as gastrointestinal disorders, weight loss as well as more severe paresthesia, polyneuropathy, peripheral-nerve inflammation, bone-marrow dysfunction, and heart failure were reported [125,126,127,128,129]. The use of nanotechnology can have a significant impact on improving biopharmaceutical properties of the above-mentioned drugs. These features include increase in solubility, permeability, stability in the host’s bloodstream, bioavailability, and extend release time. Benzimidazole also has an anti-inflammatory effect. It is one of the main drugs used in the treatment of acute, early, and chronic Chagas’ disease, but it also causes systemic toxicity [130]. Nanosystems can help to transport the drug to the intracellular target and to reduce side-effects. This is achieved by increasing the effectiveness and bioavailability of the active compound against the pathogen, by extending the release and, thus, increasing the therapeutic index [123,131,132]. In the case of *Trypanosoma*, gold and silver nanoparticles have promising antiparasitic properties. This has been determined using both in vitro and in vivo settings [133]. However, in case of *Trypanosoma congolense* and *Trypanosoma evansi*, gold nanoparticles were less effective, with an average 50% reduction of parasite growth. Silver nanoparticles were found to reduce the growth of *Trypanosoma congolense* and *Trypanosoma evansi* by 70%, indicating that these nanoparticles might have more clearly defined targets in parasites cells [77]. Both AuNPs and AgNP displayed strong activity against *Trypanosoma brucei*, reducing parasite viability by more than 5%. These data suggest that the activity of nanoparticles against *Trypanosoma* is strongly selective for the parasite, which may also suggest strong potential for their use in infection with *Trypanosoma cruzi* [22].

### 2.4. Toxoplasmosis

Toxoplasmosis is caused by *Toxoplasma gondii* protozoan and in immunocompetent subjects in 80% of cases is asymptomatic. In the remaining, 20% is oligosymptomatic (enlargement of lymph nodes, flu-like symptoms, pneumonia very rarely, and myocarditis). Most often, the symptoms disappear and do not require treatment as the infection takes the latent form. The preventive measures and diagnostics of toxoplasmosis are of special importance in efforts to avoid development of its congenital type, which might cause birth defects and the death of fetuses. The course of toxoplasmosis depends, partly, on the particular *Toxoplasma gondii* strain and its intensity is determined mainly by the host’s immune response [134,135]. In most cases, the *T. gondii* infection does not produce any symptoms, but the protozoan is capable of producing tissue cysts that can be present for many years. Individuals particularly susceptible to symptomatic infections are those with cancer, AIDS, or ongoing immunosuppressive treatment, in whom the infection may cause neurological, ocular, and systemic complications [136,137,138,139,140,141].

In pregnant women, the consequences of *Toxoplasma gondii* infection depend on the stage of pregnancy. Miscarriages and nervous-system malformations of the fetus occur mainly in the first trimester of pregnancy [134,140]. In subsequent trimesters, the likelihood of damaging the fetus is lower, but it is more likely that the protozoan will overcome the mother–fetus barrier. The first prevention measure to undertake should be health education to limit the spread of toxoplasmosis in the human and animal population (especially, to reduce the exposure of pregnant women who will be more likely affected by primary infection). The second measure should be based on the mass screening of pregnant women and serological tests of neonatal blood to detect fetal infection. Diagnosis of pregnant women is essential in determining the type of infection (primary, or secondary), which translates to the probability of the parasite’s penetration through the placenta. The use of direct methods allows the detection of the presence of a parasite in the diagnostic specimens, such as amniotic fluid, placenta, umbilical cord blood, and fetal blood (identification of congenital infection) [142,143,144]. Indirect methods—biological tests involving the introduction of a parasite into laboratory animals or tissue cultures—are rare due to the time-consuming nature of the process and limited accessibility. MeNPs can be used in the diagnosis of toxoplasmosis, improving the effectiveness and sensitivity of tests. One example of such a test is a dynamic imaging test (DFICT) with sensitivity and specificity of 92% and 93.1%, respectively. This test involves use of gold nanoparticles [145,146,147,148]. Using nanoparticles modified with poly methacrylic acid (PMAA) coupled to the anti-human IgM provides a test with near-100% sensitivity and specificity, with the possibility of simultaneous detection of several pathogens [146,149]. A similar test is carried out using magnetic nanoparticles (IMB-ELISA) to detect *Toxoplasma gondii* surface antigens circulating in human-serum samples with a sensitivity of 98% and a specificity of 96.4% [150]. Nanotechnology has also found application in *Toxoplasma gondii* diagnostic tests involving the detection of genetic material using DNA probes with nanoparticles [151]. From a diagnostic platform, quantum dot probes synthesized with nickel nanoparticles (CdTE/Ni mQD) with magnetic and fluorescent properties, provide high sensitivity, specificity, and effectiveness in *T. gondii* DNA sensing [147].

In prevention methods against *Toxoplasma gondii*, the usage of nanoparticles for the development of an effective vaccine represents a vital alternative. The development of vaccines is particularly important from the perspective of immunocompromised subjects. Infection in people with a properly functioning immune system is usually asymptomatic, creating the dormant forms (tissues containing bradyzoites) in the brain and muscles [152]. As a result of decreased immunity, these dormant forms may reactivate and cause widespread tissue destruction. Such reactivation of latent infection is one of the main causes of mortality in AIDS-suffering patients. Different vaccination trials performed so far in animals with inactivated/attenuated vaccines, DNA vaccines, subunit vaccines, and genetically engineered vaccines indicate that development of such protective strategies for practical use in humans is possible [153]. The possible use of vaccines in humans against *Toxoplasma gondii* is still questionable, as some experiments that are effective in animal models still fail to provide protective immunity in humans. The *Toxoplasma gondii* life-cycle is very complex, resulting in the expression of different proteins with a high degree of antigenic polymorphism and variability, which depend on host and invasion pathways. So far, only a limited number of tested *Toxoplasma gondii* antigens were found to induce long-lasting immunity. Overall, the strategy to design effective anti-*Toxoplasma* vaccines should include optimal choice of the antigens and a proper administration method combined with optimized delivery systems that, taken together, will provide efficient induction of the immune response. In this context, MeNPs might serve as adjuvant for *Toxoplasma gondii* antigens to promote activation and migration of immunocompetent cells, which are involved in antigen presentation [33].

The vaccine against *Toxoplasma gondii* should provide high Th1-type immune response and prevent the development of the chronic form of the disease. Since there are many strains of *Toxoplasma gondii* and the parasite has innate mechanisms of avoiding immunity [154,155], this requirement is of particular importance. As mentioned above, the protein subunit vaccines or polyvalent subunit vaccines (“antigen cocktails”), as well as DNA vaccines, have been tested in animal models. They are an alternative to inactivated vaccines containing subcellular components of the parasite, such as SAG surface antigens. In *Toxoplasma gondii* tachyzoites, there are three types of characteristic surface antigens (SAG1, SAG2A, and SAG2B). The same situation is found in bradyzoites, although the antigens are different (SAG2C, SAG2D, and SAG4). The SAG3 antigen is a common future in both forms of *Toxoplasma gondii*. Vaccinations with combinations of the above-mentioned antigens in mice resulted in an increased survival rate after infection with a particularly lethal type 1 parasite. Better results were obtained after using poliglikolid (lactide-co-glycolide PLG) microparticles in combination with SAG1 and SAG2 protein antigens, which promote the sustained release of the antigens [155]. Studies were performed to develop a DNA-based vaccine against *Toxoplasma gondii* ribosomal P2 protein, which had its efficacy boosted by the usage of two nanomaterials—poly-lactic-*co*-glycolic acid (PLGA) and chitosan [156]. The DNA of the vaccine was trapped inside nanospheres, which were created from these nanomaterials. Study results show that both these vaccines elicited mixed Th1–Th2 immune response in mice model. The titers of both IgG1 antibodies (associated with Th2-related immunity) and IgG2a antibodies (associated with Th1-related immunity) were higher to the titers obtained when the DNA of the vaccine was administered without nanospheres. These results are promising from the point of view of the usage of nanomaterials in boosting the immune response against parasitic invasions.

Another research was conducted using self-replicating RNA amplicons, encapsulated in modified dendrimer nanoparticles (MDNP) [157]. The replicon used encoded GRA6, ROP2A, ROP18, SAG1, and SAG2A as well as apical membrane antigen-1. The nanosystem is capable of delivering this self-replicating RNA into the cytoplasm and to protect it against the action of cellular nucleases. The administration of this nanosystem-based RNA vaccine was able to protect mice against lethal infection by *T. gondii*. Additionally, the vaccine was effective enough to activate and stimulate the proliferation of CD8+ T lymphocytes, as well as to increase the expression IFN-γ and IL-2 [158]. Promising results in the treatment of toxoplasmosis have been achieved by the usage of chitosan nanoparticles (CSNPs), which display a high anti-parasitic activity against tachyzoites of *T. gondii* strain RH. The spiramycin-loaded chitosan nanoparticles (SLCNs) were demonstrated to increase the antiparasitic effect in both acute and chronic *T. gondii* infections [159,160]. A similar result was obtained with the use of calcium carbonate nanoparticles loaded with benznidazole (BZN@CaCO_3_) on the *Trypanosoma cruzi* species responsible for the Chagas’ disease. The use of BZN@CaCO_3_ decreased the viability of epimastigote and trypomastigote *Trypanosoma cruzi*. The antiparasitic effect was achieved with a lower-than-usual concentration of benznidazole; the effective usage of such a lower concentration of the drug may contribute to lessening the possibility of drug toxicity and, thus, adverse events [161].

The efficacy of similar nanoparticles can also be demonstrated in single-chamber echinococcosis caused by *Echinococcus granulosus* in which the colloimidic activity of biosynthesized nanoparticles (AgNPs) displayed up to 90% eradication ability of protoscolexes [162]. The efficacy of gold nanoparticles (AuNPs) was tested not only in the case of toxoplasmosis, but also to fight schistosomiasis, leishmaniasis, trypanosomiasis, cryptosporidiosis, and malaria [27,65,77,163,164,165,166,167,168]. The bioactivity of AuNPs was also tested in relation to selected species of insects of public-health importance, mainly including mosquito vectors, such as *Aedes aegypti*, *Anopheles stephensi*, *Culex quinque fasciatus*, *Culex mites*, and some tick species [169]. Very good results were observed with the use of silver and platinum nanoparticles, which caused >90% inhibition of *T. gondii* growth, with limited cytotoxicity to the host cells [170,171]. More effective action of gold and silver nanoparticles can also be observed using nanosystems AuNP-TRP and AgNP-TRP (both being of NPs conjugates with tryptophan) characterized by high activity against *T. gondii*. It happens so because *Toxoplasma* is auxotrophic for l-tryptophan (normally, it acquires tryptophan from the host in order to survive). The conjugation of NP with this amino acid promotes their uptake into parasite cells, thus, making them more sensitive to such nanosystems.

Atovaquone is used for the treatment of toxoplasmosis, especially in people with HIV/AIDS, malaria, or *Pneumocystis jiroveci* pneumonia. Unfortunately, this drug has low solubility and, thus, reduced bioavailability. Solubility may be improved by incorporation of atovaquone into conjugate nanosystems, for example, those with MeNPs [172,173,174]. The testing of polymeric polylactic nanocapsules (PLA) conjugated with atovaquone in the treatment of acute and chronic toxoplasmosis (animal model using mice) showed a positive therapeutic effect and improved drug activity. This therapy has shown a higher reduction of parasite load and an increased survival rate of experimental animals when compared to free atovaquone. A similar use of nanomaterials that improve bioavailability and activity of the drug has been demonstrated in experimental treatment with resveratrol. Resveratrol has a strong anti-inflammatory and antioxidant effect, as well as antibacterial, antifungal, antitumor, and antiparasitic properties. In addition, resveratrol regulates immunity by interfering with the regulation of immune cells, the synthesis of pro-inflammatory cytokines, and gene expression. Resveratrol participates in the activation of macrophages, T cells, and natural killer (NK) cells, and is involved in the suppressive regulatory functions of CD4 + CD25 + T cells. Pharmacokinetic analysis reveals that resveratrol is rapidly metabolized in the body and its oral bioavailability is very low, despite its absorption reaching 70%, which is associated with the use of high-dose concentrations that may cause immunosuppression [175]. To overcome these issues, resveratrol had been complexed with 2-hydroxypropyl-β-cyclodextrin (HPβCD). These complexes could also be additionally bound to sulphamethoxazol-trimethoprim particles. Treatment with such drug combinations reduced the number of brain cysts and inflammatory infiltrates in experimental animals—both in species infected with tachyzoites and bradyzoites of *T. gondii*. Such treatment had no effect on creatine kinase activity—an enzyme associated with energy metabolism of the brain—which proved better protection by resveratrol against inflammation within the CNS [138].

The promising use of nanotechnology in toxoplasmosis treatment might be expected with compounds such as ceragenins, which display a very broad antimicrobial spectrum of activity. The results of studies involving ceragenins CSA-13, CSA-44, CSA-131, and CSA-138 against the sensitive and metronidazole-resistant *Trichomonas vaginalis* protozoans allowed for an assumption, that these substances might be used against intracellular forms of *Toxoplasma gondii*. The application of metallic nanoparticles along with ceragenin as a core-shell nanosystem may result in mutual reinforcement of their biological action in relation to intracellular forms of *Toxoplasma gondii* [176]. The desired actions, i.e., the ability of drugs to cross biological barriers and to eliminate specific forms of this parasite (while minimizing side-effects) has yet to be achieved, but the likelihood of obtaining such therapeutic tools increases with the development and use of nanotechnology, as indicated in Table 3.

### 2.5. Malaria

Malaria is responsible for approximately 1.5 to 2.7 million deaths per year. Malaria is caused in humans by intra-erythrocyte protozoa of the *Plasmodium* species, which are transmitted by the *Anopheles* mosquito species. Of the *Plasmodium* species infectious in humans, *Plasmodium falciparum* and *Plasmodium vivax* are the most common malaria parasites in Africa and most of Asia. *Plasmodium falciparum* is responsible for cerebral malaria and the overwhelming majority of malaria deaths [181]. New methods for malaria diagnostics were developed using nanotechnology approach. A quantum-magnetic droplet test (MB—QD) allows for detection of *Plasmodium falciparum* protein 2 (HRP2—protein 2; rich in histidine) in serum or urine. Magnetic beads in combination with the anti-HRP2 antibody allow for the capture of target protein, which is then concentrated and detected using quantum dots [182]. For successful treatment of malaria, the main therapeutic option is artemisinin-based combination therapy (ACT)—currently one of the most effective antimalarial drugs, including the recommended mainstay in the treatment of *Plasmodium falciparum* infection. In addition, chloroquine and tafenoquine are used (please see Table 1), but the parasite shows resistance to these compounds in an increasing number of infections [183].

Gold and silver nanoparticles showed high activity against drug-resistant parasites [91]. The use of metallic nanoparticles in the treatment of malaria is supported by the fact that they can be synthesized according to the methods of “green chemistry”—using plant extracts such as those obtained from *Andrographis paniculata* or *Calotropis gigantea*. The plant extract might have its antiparasitic activity increased due to the presence of biological compounds, such as flavonoids, phenols, terpenes, steroids, and phenolic acids [184,185,186]. Some metal-oxide nanoparticles also have a strong anti-malaria effect. Oxide nanoparticles, such as Fe_3_O_4_, MgO, ZrO_2_, Al_2_O_3_, and CeO_2_, were coated with PDDS and their activity against *Plasmodium falciparum* was assessed. It was shown that PDDS-coated nanoparticles exhibit significantly better antimalarial activity than uncoated nanoparticles. Several researchers have demonstrated the anti-malarial effects of silver nanoparticles [76].

Studies have shown that photosynthetic nanoparticles have a lethal effect on different stages of malaria vectors. The best effect was observed in the use of gold and silver nanoparticles, especially in the larval and adult stages (mosquitoes egg are less susceptible to silver and gold nanoparticles) [184].

### 2.6. Schistosomiasis

Schistosomiasis is a very common parasitic disease. In humans, it is caused by the invasion of cercariae through the skin, usually during swimming in contaminated water. It is common in tropical and subtropical regions, mainly in places where safety and health-protection measures are limited. It is estimated that about 600–770 million people are at risk of being infected and that the disease currently affects over 200 million people worldwide. The estimated mortality in Sub-Saharan African countries is about 280,000 deaths per year [63].

The symptoms of schistosomiasis include abdominal pain, hematomas, anemia, and fibrosis of the bladder and liver. Currently, there is no vaccine against schistosomiasis, and treatment is mainly conducted with the use of the antihelmintic drug Praziquantel (PZQ), as indicated in Table 1. This drug is not effective against immature forms of the *Schistosoma* species and its misuse has led to the development of drug-resistant *Schistosoma* variants in many regions around the world [63].

To minimize the incidence and achieve the elimination of the pathogen, early diagnosis is the primary objective. Developing new ways of detection of *Schistosoma*, as well as observation of the progress of the disease, play a very important role.

Electrode biosensors are sensors that can capture trace amounts of analytes by detecting changes in electric current, electric potential, or conductivity caused by an immune response. They allow for significant differentiation in the shape of electrodes and material selection. The connection of nanomaterials to these sensors allowed for the increase in their sensitivity in detection of biomolecules at relatively lower thresholds. Biosensors based on nanotechnology (nanosensors) seem to be exceptionally sensitive, which is a promising solution in medical diagnostics. Modified biosensors are composed of various nanomaterials, i.e., carbon nanoparticles with gold and silver attached, magnetic nanoparticles, or carbon nanotubes. Nanomaterials can also be used to increase sensitivity and improve antigen- and antibody-based detection methods. So far, most studies have been based on the detection of the *Schistosoma* antigens. The example is an enzyme affinity test (magnetic affinity enzyme-linked immunoassay—MEIA). The MEIA test is based on the production of specific antibodies in response to the appearance of worm or larval antigen. In the MEIA test, magnetic beads have superparamagnetic nanoparticles and functional polymer materials. Additionally, magnetic beads were transformed by means of characteristic functional groups to be able to bond with charged molecules. The magnetic field was applied to the reactive system after setting molecular targets on magnetic beams. As a result, an immune complex was obtained by magnetic separation. In another diagnostic method, colloidal gold, a different variant of metallic nanoparticles was used. Thanks to the possibility of binding with biological macromolecules, i.e., immunoglobulins, such Ig-bonded colloidal gold is a practical device for key studies on clinical diagnostics. During the diagnostics process, schistosomal antigen produces an immune complex with IgG–colloidal gold [88].

As mentioned above, Praziquantel is active against all species of *Schistosoma*. It causes morphological changes in the parasite’s coating which increases antigen exposure to the action of the immune system. It also affects muscle contraction—probably through a rapid inflow of Ca^2+^ ions. In the past, some other drugs were used (metrifonate, oltipraz, niridazole, and oxamniquine). All of them were withdrawn due to high toxicity, carcinogenicity, and lack of desired efficacy against the parasite [63]. Facing so many flaws of current therapies, the use of nanotechnology in the precise delivery of the drug seems to be an appropriate approach. The main goal is the selection of appropriate nanoparticles for the delivery system in order to minimize side-effects and improve the effectiveness of the selected drug. This is particularly important in cases of co-infection with several strains of *Schistosoma* or in cerebral schistosomiasis. During the development of novel treatment regimens against schistosomiasis, the following nanosystems are used: liposomes, micelles, polymer nanoparticles, and metallic nanoparticles. Among the metallic nanoparticles, mostly silver and gold nanoparticles are used. It has been observed in *Schistosoma mansoni* infection (tested in mice model) treated with different doses of AuNPs, that nanoparticles have a beneficial effect on oxidative stress caused by the disease. AuNPs also alleviate inflammatory cell infiltration, reduce the diameter of granules, as well as reduce the extent of histological damage in the central nervous system [87].

## 3. Immune Response during Host—Parasite Interaction. Can MNPs and MNPs-Containing Nanosystems Be Used as Immunomodulators during the Course of Parasitic Diseases?

The host’s immune response to the parasitic infection depends mainly on the interaction of the parasite and the host’s immune system. The host defense mechanisms are usually not effective enough to eradicate the invading parasite. This allows the parasites to reach their target organ, therefore, gaining time for progression of the disease and development of the parasite itself [187]. 

The host’s immune response to the parasitic infection may be immediate characterized by significantly increased eosinophilia, and mast cell and basophil tissue infiltration. High levels of IgE antibodies were also observed (several times over the norm) as well as the adaptation process of T CD4+ lymphocytes [188,189,190]. These cells participate in the Th2 pathway of the immune system and can synthesize IL-4 cytokine that participates in starting physiological reactions to parasitic infections. The Th2-pathway-associated cytokines include interleukins production, including of IL-4, IL-13, IL-5, IL-9, IL-10, IL-25, and IL-31 [191]. During parasitic worm infections, the development of the Th2 response is observed, while during protozoan infections (e.g., in the *Toxoplasma gondii* invasion), the development of the Th1 response is reported. The main means of defense against intracellular parasites is the cellular response in individuals with properly functioning immune system. In the acute phase of the invasion, as well as in the chronic stage of the disease, CD4+ and CD8+ lymphocytes kill the parasite-infected cells (CD8+ also kill the extracellular forms), and they contribute to the development of immunity against *Toxoplasma gondii* through cytokine production [192]. Th1 lymphocytes producing IFN-γ, IL-2, TNF-β, and IL-12 are responsible for the defense against the intracellular forms, while the Th-2 response (characterized by the production of IL-4, IL 5, IL-6, IL-9, and IL-6) serves for strengthening the humoral reactions and destruction of the extracellular forms. So far, we do not have strong experimental data indicating the possibility of enhancing the antiparasitic immune response with metal nanoparticles. However, such a possibility may be suggested based on the physicochemical properties of metal nanoparticles coated with molecules derived from parasites, which stimulate the host’s immune response, allowing for their high local concentration based on an optimized surface-to-volume ratio within synthetized nanosystems.

## 4. Parasitic Invasion of Host Cells as Potential Targets for Nanosystem Action

In this article, the strategy based on the blocking of the parasitic invasion of host cells is illustrated with the examples of two parasites caused by *Plasmodium* and *Toxoplasma gondii*. Malaria in humans is caused by five *Plasmodium* species—*Plasmodium falciparum*, *Plasmodium vivax*, *Plasmodium ovale*, *Plasmodium malariae,* and *Plasmodium knowlesi*—and is a disease with acute symptoms, ranging from recurrent fever, headache, muscle and joint pain, vomiting, jaundice, and anemia, leading to serious complications, such as acidosis, respiratory failure, kidney failure, and cerebral malaria [193]. Parasites are located only in erythrocytes and, thus, theoretically, can reach all host tissues through circulation. The parasites (a form of merozoite) are released into the bloodstream from hepatocytes enveloped in merosomes and continue a repetitive cycle of erythrocyte invasion. The infection of erythrocytes causes their extensive remodeling and, thus, dysfunction, followed by cell lysis, which contributes to the development of symptoms such as severe anemia. The merozoite entry is a complex and dynamic process in which the apex end of the merosome must be directed towards the erythrocyte, where specialized secretory organelles (called the micronemes, rhoptries, and dense granules) are located. When the merosome is connected to the erythrocyte, its double layer is opened, and the parasite (merozoite) is absorbed. Once inside the erythrocyte, the parasites mature and, due to their protein production, remodelling of the cells can occur. The parasite proteins are released after erythrocyte destruction; these proteins interact with uninfected erythrocytes to change their antigen and vascular properties. As a result of this process, infected as well as non-infected erythrocytes are remodelled by the parasite which initiates their removal by the spleen. This process may exacerbate anemia [194]. Repeated cycles of erythrocyte invasion lead to the release of the parasite, deepening of anemia, toxic hem removal, activation of immune cells (thrombocyte modulation, neutrophilia, monocytes, macrophages, T cells, and B cells), and may cause a cytokine/chemokine storm to develop. The knowledge of repetitive cycles of erythrocyte invasion in malaria processes became a base for the development of a very sophisticated strategy of usage of nanomaterials to prevent *Plasmodium falciparum* entry into the host’s red blood cells [195]. The nanomaterials were named nanomimics, and two co-polymers were used to create it: polymersome-forming ABA block copolymer poly(2-methyl-2-oxazoline)-block-poly(dimethylsiloxane)-blockpoly(2-methyl-2-oxazoline) (PMOXA-b-PDMS-b-PMOXA) as well as the PDMS-heparin block copolymer. These nanomimics contained cellular receptor analogues for *Plasmodium falciparum* and were shown to efficiently block the entrance of the parasite into host red blood cells (as compared to soluble receptor proteins). The authors suggest that this blockade of entry can leave the parasite vulnerable to both the host’s immune response and the action of co-administered anti-plasmodial therapeutics.

In zoonosis caused by the protozoan *Toxoplasma gondii*, the parasite has the ability to actively penetrate and establish itself in most host cells, forming a parasitic vacuole. The protozoan affects the apoptosis process and induces changes in the host’s immune response. After penetration into the host, the parasite proliferates rapidly in the cells of the reticuloendothelial system (phagocytic system) and in the enterocytes that build the small intestinal mucous membrane, where it forms the pseudocysts containing many daughter cells (tachyzoites). Host enzymes lysozyme and hyaluronidase participate in the penetration of protozoan into these cells. Then, through blood or lymph (inside monocytes and neutrophils), the parasite is transported through the body. As a result of subsequent divisions, the destruction of host cells takes place and tachyzoites are released, which results in subsequent cell invasion and destruction, causing inflammatory and necrotic changes of tissues and organs. People with a properly functioning immune system, experience the acute phase to change into a chronic phase as a result of the immune response; in the chronic phase the tissue cysts with bradyzoites are formed. The cysts can develop in nearly all host cell types but most frequently they are formed in the nerve tissue (the central nervous system and retina neurons), striated muscle cells, and the cardiac muscle.

**Figure 2:**
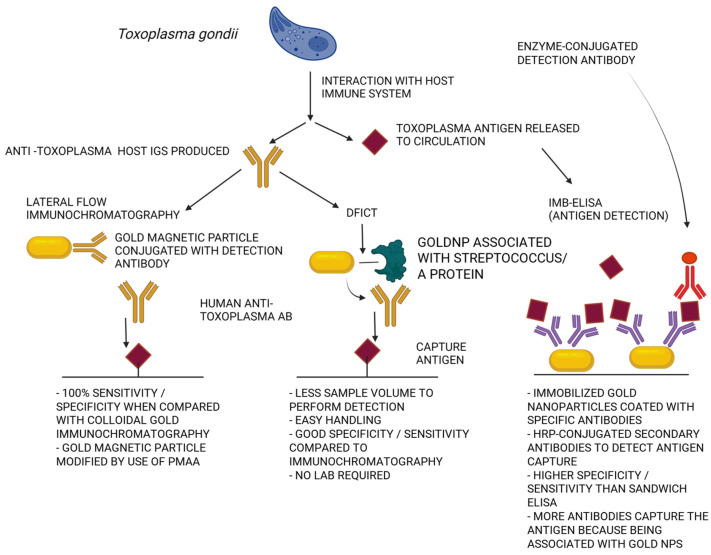
Possible enhancements of current immunological assays by the introduction of nanoparticles.

Overall, before these processes can begin, the protozoan must be positioned properly in relation to the affected cell (the so-called apical pole). In adhesion, the key component is the extracellular matrix lamina, which supports the *Toxoplasma gondii* cell. The presence of the lamina receptors on the parasite cell surface partly explains the possibility of infecting almost all the nucleated host cells. The lectins and the surface antigens from the SAG family (mainly SAG 1 protein) located on the surface of *Toxoplasma gondii* cells are also involved in the adhesion process. After reaching the proper location on the host cell and after recognition of surface receptors by SAG proteins, the concentration of Ca^2+^ ions in the parasite cell increases. This initiates the increase in the production of soluble proteins—micron (MIC). The parasite at the apical end of its cell has numerous secretory organelles (micronemes) from which the secretion of many MIC proteins takes place. The proteins mediate the invasion process; they are intended to stabilize the process of creating a parasitic vacuole, which is located in the apical end of the cell. The parasitic vacuole is a separated compartment within the host cell in which the parasite multiplies. The accumulation of proteins contributes to the formation of two adhesive complexes: MIC6-MIC1-MIC4 and MyoA complex (called A motor complex)—built of MIC2 and M2AP proteins that bind to aldolase, binding also the actin filaments. Then, as a result of the transformation, the cells of the parasite release the proteins with AMA1 and the proteins originating from the roptria neck (RON) create stable complexes that build the structure of the moving junction (MJ). ”Insertion” of the parasite cell depends on movement of this complex from the apical pole to the opposite pole with the participation of myosin. When the entire cell passes through the MJ structure, the outer membrane closes to form a parasitic vacuole, in which subsequent tachyzoites develop. The MIC6-MIC1-MIC4 is an adhesive complex containing the so-called “escorting protein” responsible for the correct access to the micron. MIC1 is the core of the complex—it is defined as a lactose-specific lectin which has the ability to bind glycoprotein receptors containing lactose, occurring on the surface of the attacked host cell. MIC 6 functions as an anchor of the adhesion complexes. MIC 2 companion proteins and M2AP bind to aldolase; this enzyme creates a bridge between them and the actin fibers of target cells. This complex, with the help of myosin, initiates the process of the parasite “slipping” into the host cell. The TgMyoA (myosin A motor complex) is comprised of micron proteins MIC 2 and M2AP, which are associated with aldolase binding to the actin pillars [196,197]. Different kinds of nanoparticles can affect the *Toxoplasma* ability to enter the host cells. AuNPs decrease the ability to invade host cells by >65%, AgNPs and PtNPs by >60% and >50%, respectively. At the same time, the nanoparticles reduce parasite replication in vacuoles by >67%. Probably, these anti-parasitic effects are caused by NPs’ stimulation of the host cells rather than by directly affecting the parasite itself. These anti-parasitic effects may be associated with the NPs’ ability to induce redox potential of the host cells (see Figure 2).

## 5. Challenges and Prospects for Future Use of Nanomaterials against Human Parasites

Metallic nanoparticles are considered to be effective as delivery systems for antiparasitic drugs. Currently, there is still a lack of such combined drugs on the market, but there are still many of them being subjected to studies, as well as preclinical and clinical trials. Although the use of nanoparticles offers great potential for the delivery of anti-parasitic drugs, there are still several limitations to overcome. Nanoparticles need to have increased absorption, prolonged release time, proper intracellular delivery systems, proper therapeutic effect, and should be affordable and safe to use. Unfortunately, artificially synthesized nanoparticles might be toxic, so it is important to continue research on new biocompatible and biodegradable nanomaterials. The combination of metallic nanoparticles with plant extracts has also resulted in obtaining better tools against parasitic worms. MeNPs have unique properties, such as electrical conductivity, high thermal and chemical stability, and the ability to transform into bioconjugates. Properly selected particles can effectively deliver drugs (which show high drug stability), reduce toxicity, have targeted affinity, controlled release, and safe degradation. Overall, research on the use of metallic nanoparticles in parasitic diseases is still relatively insignificant when compared to similar studies concerning other infectious diseases. There is clearly a need to create new nanoparticles and new nanosystems for safe and effective antiparasitic drug delivery. It is also crucial to study the toxicity and pharmacokinetics of metal-based compounds in the experimental setting related to pathophysiology of parasitic diseases.

## Figures and Tables

**Table 1:** Selected drugs used against parasites.

Group of Antiparasitic Drugs	Antiparasitic Drugs
Anti-malarials	artemisinin chloroquine, amodiaquine, atovaquone, primaquine, mefloquine, atovaquone-proguanil, doxycycline
Anti-babesial agents	atovaquone, azithromycin, clindamycin, quinine
Anti-amoebic agents	iodoquinol, paromomycin sulfate, diloxanide furoate, metronidazole, tinidazole, emetine, chloroquine
Anti-giardial agents	metronidazol, tinidazole, furazolidone, albendazole
Trypanocidal agents	nifurtimox, benznidazole, pentamidine, eflornithine, suramin, melarsoprol
Anti-leishmanial agents	meglumine antimoniate, pentamidine, amphotericin B
Anti-toxoplasma agents	sulfadiazine, pyrimethamine, sulfamethazine, sulfamerazine
Anti-cestodal drugs	praziquantel, niclosamide, albendazole
Anti-nematodal drugs	praziquantel, metrifonate, oxamniquine Albendazole, diethylcarbamazine, ivermectin

**Table 2:** Selected studies on metal-based nanoparticles used in eradication of parasites.

Species of the Parasite	Nanoparticles	Therapeutic Result	Bibliography
*Trypanosoma* *brucei*	AgNP, AuNP, PtNP	strong anti-parasitic effect	[77]
*Leishmania* *tropica*	ZnONP	effect on the cell membrane of the parasite	[78]
*Leishmania* *major*	ZnONP	induction of apoptosis, toxic effect on the promastigote of *L. major*	[79]
	AgNP, AuNP, TiO_2_NP, ZnONPiMgONP	high antiparasitic activity	[80,81,82,83,84]
*Leishmania* *donovanii*	ZnONP	cytotoxic effect on the amastigote	[85]
*Schistosma* *mansoni*	AuNP	antihelmintic action	[63,86,87]
	carbon nanoparticles with gold and silver, magnetic nanoparticles, carbon nanotubes, colloidal gold	high anti-parasitic activity	[86,88,89]
*Acanthamoeba* *keratitis*	AgNP	anti-amoebic effect on *Acanthamoeba* trophozoites	[90,91,92,93,94,95]
*Plasmodium* *falciparum*	metal oxide nanoparticles Fe_3_O_4_, MgO, ZrO_2_, Al_2_O_3_, CeO_2_, AuNP	demonstrated anti-plasmoid activity	[65,91]
*Toxoplasma* *gondii*	AuNP AgNP	high anti-parasitic activity	[96,97]

**Table 3:** Examples of nanoparticles and nanosystems used in diagnostics and therapy of *Toxoplasma gondii* infection.

Nanoparticles and Nanosystems Used in Management of Toxoplasmosis	
in Diagnostics:	in Therapy:
Nanoparticles of zinc oxide (ZnO-NP) covered with chitosan [149]	Chitosan and silver nanoparticles [171]
Silicon coated nanoparticles (SiO2NP) [150]	Curcumin nanoemulsion [177]
Gold nanoparticles [178,179]	Gold and iron nanoparticles [33] Lipid-core nanocapsules with pyrimethamine [180]

## Data Availability

Not applicable.
